# The Influence of Family Sports Attitude on Children’s Sports Participation, Screen Time, and Body Mass Index

**DOI:** 10.3389/fpsyg.2021.697358

**Published:** 2021-12-20

**Authors:** Yin Lian, Chen Peijie, Wang Kun, Zhang Tingran, Liu Hengxu, Yang Jinxin, Lu Wenyun, Luo Jiong

**Affiliations:** ^1^Research Center for Exercise Detoxification, College of Physical Education, Southwest University, Chongqing, China; ^2^Chongqing Medical and Health School, Chongqing, China; ^3^Leisure College, Shanghai University of Sport, Shanghai, China; ^4^Integrative Exercise Physiology Laboratory, Department of Physical Education, Jeonbuk National University, Jeonju, South Korea

**Keywords:** children, family sports attitude, parents’ education level, body mass index, screen time

## Abstract

**Background:** Children’s physical health is an important resource for a country’s future construction. However, researchers found that the physical fitness of young children around the world has declined during the two decades, from 1992 to 2012. The decline in the physique of young children has caused widespread concern around the world. Children’s main living places are families and kindergartens, so this research explores the impact of family factors on children’s body mass index (BMI) from the perspective of family attitudes, children’s sports participation, and screen time.

**Methods:** A cross-sectional study was used to conduct a questionnaire survey of children in China. A total of 600 children were investigated, and 589 valid questionnaires were obtained. SPSS21.0 statistical analysis software was used for descriptive analysis, mean comparison, and correlation analysis of the data. AMOS 21.0 was used to construct a structural equation model and carry out path analysis.

**Results:** (1) There are significant differences in children’s family sports attitude, sports participation, screen time, and BMI with different family structures, and parents’ education levels. (2) Family sports attitude is significantly positively correlated with parents’ education levels and children’s sports participation, and negatively correlated with children’s screen time and BMI. (3) Children’s sports participation and screen time play a chain-mediating role between family sports attitude and children’s BMI, and the role is a complete mediating role. Therefore, family sports attitudes can affect children’s physical health by affecting children’s sports participation and screen time. To promote children’s physical health, we should pay attention to the intervention of family sports attitude. (4) The mediating effects of exercise participation and screen time are similar in different family structures, so the structure of this study can be applied to different family structures.

**Conclusions:** Children’s family sports attitude, sports participation, and screen time can affect children’s BMI. Children’s screen time and sports participation play a chain-mediating role in the influence of family sports attitudes on the path of children’s BMI. The results of this study will provide a useful reference for teachers and parents to control children’s physical health.

## Introduction

Children are the fundamental guarantee of human sustainable development^1^. As an important resource for the future construction of a country, their physical health is a strategic issue directly related to the future and destiny of the country (Liu et al., 2021). Children’s physical fitness has always been a health issue of global concern. A survey of children and adolescents aged 2–19, including toddlers, showed that obesity rates rose by 6.9% for males and 6.4% for females in developed countries; obesity rates in developing countries rose by 4.8% for males and 5.0% for a female between 1980 and 2013 (Ng et al., 2014). According to the 2014 National Physical Fitness Monitoring Survey, the level of morphological indicators such as height has improved in China, while the level of physical fitness has decreased, ranging from 0.1 to 2.3%. The Report on Childhood Obesity in China points out that the obesity rate of children in large- and medium-sized cities alone is 4.3%. Without effective intervention measures, the obesity rate of children will rise to 6% by 2030, which seriously threatens children’s physical health. Therefore, it is of great significance to pay attention to children’s physical quality for the sustainable development of children’s physical health (Pooja et al., 2016; Zhang et al., 2017). Since 2000, by the national physical fitness monitoring indicators issued by the state, China has conducted regular and unified tests on the monitored objects nationwide in the form of sampling surveys; children aged 3–6 are the important monitoring objects. The age of 3–6 is a critical period for children’s growth and development. A follow-up investigation found that obesity in early childhood can increase the probability of obesity in adulthood (Freedman et al., 2005; Singh et al., 2008). And obesity can cause chronic diseases, such as hypertension, diabetes, and cardiovascular disease (Hu et al., 2004; Reaven, 2011). Therefore, it is of great significance to investigate and monitor children’s family environment, sports behavior and shape, quality, function, and other content indicators at this stage (Zhang et al., 2019).

Among physical measurement indexes, Body Mass Index (BMI), which is calculated as BMI = weight (kg)/height (m)^2^, is an objective index commonly used in the world to measure the degree of body fat and thinness (Dietz and Robinson, 1998). BMI, as one of the important indicators for child nutrition monitoring and obesity screening, has reached broad consensus on theory and practice. Many organizations and countries around the world have established the standard curve of BMI percentile and the threshold of overweight and obesity screening for preschool children, such as the International Obesity Organization (IOTF) (Cole and Lobstein, 2012), WHO (de Onis et al., 2007). Relevant research found that BMI was not only significantly correlated with the occurrence of hyperglycemia, hypertension, hyperlipidemia, and other diseases but also can directly reflect the body fat level of the human body. This index was also significantly correlated with the health of children and adolescents after adulthood (Carson et al., 2016, 2017). Due to their cognitive characteristics, children’s cognitive ability is lower than that of adults. Generally, children cannot rely on direct experience when acquiring information, and they need to rely more on people around them to provide information. Studies have found that parents’ upbringing, static screen time was too long, and inadequate activity would lead to children’s physical deterioration, and parents’ upbringing was the integrated embodiment of its education behavior and idea, which also was a kind of performance in the process of child-rearing behaviors and tendency; beyond that, family structure and the cultural level can significantly affect their parents’ upbringing (Zhou et al., 2014; Lu et al., 2015; Chen et al., 2020). Although there are many studies on the influence of the family environment on the next generation’s sports healthy behavior, previous studies mainly focused on the influence of the family capital on its healthy behavior. Moreover, very few studies focused on the influence of family sports attitude on their sports behavior, screen time, and BMI. In addition, previous studies on children’s BMI mainly focused on descriptive analysis without effectively exploring its path relationship. Therefore, the path relationship between the influence of family sports attitude on children’s BMI, especially in different family structures, remains to be further studied. Therefore, based on the analysis of previous studies and by the characteristics of children’s growth and development, this study constructed a theoretical framework model of family sports attitude, affecting children’s BMI. This study explored the influence mechanism of family sports attitude on children’s BMI.It is found that family sports attitudes can affect children’s physical health by influencing children’s screen time and sports participation. The results of this study will provide a useful reference for the rational design and planning of children’s physical and mental health courses, and it will provide a useful reference for improving children’s physical activity level and preventing children’s obesity. Therefore, based on the analysis of previous studies and by the characteristics of children’s growth and development, this study constructed a theoretical framework model of family sports attitude affecting children’s BMI, thus providing a useful reference for the future design and planning of children’s physical and mental health courses, promoting their physical activity levels and the prevention of children’s obesity in the future.

## Literature Review

### Family Sports Attitudes With Children’s Physical Activity and Screen Time

Social psychological studies have proved that values affect attitudes and attitudes affect behaviors. Sports attitude is an outer variable, which can only affect children through sports behavior. The characteristics of children’s physiological cognition lead to the need for nurturing children to provide them with sports experience and information, and sports are highly infectious. Researchers have found that children from families with supportive attitudes toward physical activities were significantly better than those who did not (Dong et al., 2018). Therefrom, family sports attitude would have a certain influence on children’s physical activity participation behavior and psychology (Timperio et al., 2013). It was found that children were more affected by the feedback of parents’ sports evaluation, and they were more susceptible to the influence of peers in adolescence. From that, parents play a key role in the influence of children’s physical activity behavior in early childhood (Fu and Li, 2004; Brown and Larson, 2009). Compared with children without parental support, children with parental support were more likely to participate in sports activities and believed that they have the better athletic ability (Budde et al., 2008; Best, 2012). A study that followed daily activities of 155 children aged 4–7 for 3 years found a high correlation between low levels of gaming activity and high BMI, while the high level of physical activity was positively correlated with the BMI-Z score in preschoolers (Foster et al., 2018; Hoare et al., 2019). The BMI of children with moderate to high-intensity physical activity was significantly lower than that of children with low-intensity physical activity (Thivel et al., 2013; Beaulieu et al., 2016). However, there are also inconsistent results with the above studies. Researchers conducted a randomized controlled intervention experiment on children in 36 nurseries in Douglas, Scotland, three times a week, and found that the BMI of the children in the low-intensity physical activity experimental group was not lower than that of the control group. However, there was a significant difference in BMI between the moderate-to-high-intensity group and the control group, so the amount of moderate-to-high-intensity physical activity would significantly affect the BMI of children (Reilly et al., 2006). Fitzgibbon et al. (2004) found that moderate to high-intensity hip-hop was effective in reducing subsequent increases in BMI in preschoolers in Chicago, Illinois, but had no significant effect in Latin America. In addition, sedentary behavior has also attracted a lot of attention from researchers, especially since the screen time of sedentary behavior has almost become the focus (Thivel et al., 2013). The survey found that the average screen time of primary school students in Hong Kong was 4 h per day on weekdays and 6 h on weekends there was a positive correlation between screen time and BMI among children (Shi et al., 2020). Three-year-old children with more than 8 h of screen time per week had an increased risk of obesity at the age of 7, a finding found in another study, which also reduced BMI, triceps sebum thickness, and abdominal circumference when screen time was reduced (Van Ekris et al., 2016).

### Parents’ Education Level and Children’s BMI

As the core of the family and the first teacher of children, the influence of parents on their children’s behavior and attitude is the focus of scholars’ research. Parents’ education levels are an important background characteristic that affects children’s development. A large number of studies have reported that parents’ socioeconomic status is closely related to their children’s cognitive ability, social emotion, and physical health. The indicators of social and economic status include parents’ education levels, income, occupation, etc., among which parents’ education levels are a stable and important indicator (Bradley and Corwyn, 2002; Matthews and Gallo, 2011). Cheng et al. (2020) found that a mother’s occupation and length of education had a significant impact on the BMI of her children. The higher her occupational class and status, the longer her education, the lower the BMI of her children. The more time children spent in physical activity with their parents, the lower their BMI was (Cheng et al., 2020). In the study of family social economy and BMI of children and adolescents, Shi et al. (2021) found that the education level of parents can significantly affect the BMI of children and adolescents, and the higher the education level of parents, the lower the probability of the children’s BMI being obese. In consequence, the mechanism of the parental educational level in the influence of family sports attitude on children’s BMI needs to be further studied.

### Family Structure and Children’s BMI

Early childhood is a critical period for the development of physical health. When children choose physical activities at a young age, they can only complete them under the guidance of caregivers, so caregivers play a pivotal role in this process, and the family environment in which children grow up and its composition are of great importance. The researchers found that the physical activity style of the caregivers, the degree of support and supervision for their children’s physical activity, and the parenting methods would all affect children’s sports participation (Zhang et al., 2017). Therefore, the composition of family members and the time and the way of accompanying children in their growth process have a significant impact on children’s sports participation and eating habits, and these factors are related to children’s BMI. There are mainly nuclear families living with their parents and non-nuclear families, such as single-parent families, intergenerational families, and three-generation families. Children with different family structures have different primary caregivers, and children’s sports participation and other factors are different due to different parenting styles and health concepts. In recent years, the influence of family structure on children’s health behaviors has gradually attracted the attention of scholars. However, the focus is mainly on the mental health problems caused by intergenerational rearing and single-parent families, while the influence of family structure on children’s physical health and sports behaviors is rarely studied (Basterfield et al., 2011). Then, the specific mechanism of children’s health differences caused by family sports attitude is still unclear, and the influence of different family structures on children’s physical fitness needs to be further studied.

In conclusion, first, previous studies have confirmed that family sports attitude has a certain influence on children’s physical activity and screen time. Second, exercise participation and screen time were important factors affecting children’s BMI. However, whether exercise participation and screen time have a mediating effect on the influence of sports attitude on children’s BMI has not been verified. Third, since the family structure can influence children’s sports behavior, it has not been confirmed whether the influence path of sports attitude on children’s BMI will change in different family structures, that is, nuclear family and non-nuclear family. Because of this, this study hypothesized that H1: family sports attitude was negatively correlated with infant BMI; Hypothesis H2: children’s activity participation and screen time played a mediating role in the relationship between family sports attitude and children’s BMI.

## Materials and Methods

### Participants

The sample was 589 children aged 3–6 years (mean = 4.55, SD = 1.16), 49.9% of girls, participated in this study with the permission of their guardians. Table 1 summarizes the demographic characteristics of the participants.

**TABLE 1:** Demographic characteristics of children (*N* = 589).

	Variable	M ± SD/n (%)	Home environment -related data		M ± SD/n (%)	
Age (years)		4.55 ± 1.16	Father’s (Mother’s) height (m)		1.704 ± 5.14 (1.59 ± 4.67)	
Body height (m)		1.07 ± 8.21	Father’s (Mother’s) Bodyweight (kg)		75.35 ± 21.99 (59.84 ± 18.29)	
Bodyweight (kg)		18.35 ± 3.16	Father’s (Mother’s) educational level	Junior high school and below		228/38.7%(214/36.3%)
BMI		15.86 ± 1.57		Senior high school (including technical secondary school		153/26.0%(90/15.3%)
Gender	Boy	290 (49.90%)		College or above		208/35.3%(285/48.4%)
	Girl	289 (49.70%)				
Family structure	Single parent	20 (3.40%)				
	Nuclear family	327 (56.30%)				
	Grandparenting	39 (6.7%)				
	Three generations under one roof	187 (32.2%)				

### Instrument

The instrument of this research was a questionnaire, which was designed and based on a large number of research literature; the following three parts were sorted out and formed:

#### Personal Background

The content includes children’s gender, age, height, weight, and other actual basic information, and the corresponding BMI is calculated according to their height and weight.

#### Children’s Sports Participation

In this part, the exercise participation calculation formula used by Fox (1999) was adopted: exercise participation = exercise frequency × (average exercise intensity + exercise duration). The higher the value, the higher the exercise participation degree. This part includes the number of days for children to have screen activities in a week, the number of times of exercise in a week, activity intensity, and activity time, etc. Specifically, the classification of exercise time is as follows: 1 means 30 min or less, 2 means 31–60 min, and 3 means 61–90 min, 4 means 91–120 min of exercise, 5 means 121 min or more. Exercise intensity is also divided into five levels: 1 means not tired at all, 2 means not tired, 3 means a little tired, 4 means very tired, 5 means very tired. The frequency of exercise is the number of exercises per week: 1 means less than one time per week, 2 means one to two times per week, 3 means three to four times per week, 4 means five to six times per week, and 5 means more than six times per week.

#### Home Environment

This part includes the basic information of children’s main caregivers, occupation, education level, physical exercise frequency, and attitude toward sports. The physical exercise attitude scale contains nine items, such as Do you think your child’s physical fitness is enhanced by playing sports games. The scale was quantified by a Likert five-point scale, according to the options “disapprove, not very agree, general, somewhat agree, and strongly agree”; the scale was rated as 1–5 points, respectively. The higher the total score, the more support the parents have for children’s sports. The pretest of the scale showed that the retest reliability was high, and the overall Cronbach α coefficient was 0.87. The overall verification results of the measurement model were as follows: χ2/df = 1.84, RMSEA = 0.03, AGFI = 0.98, CFI = 0.99, TLI = 0.99, IFI = 0.99, GFI = 0.99, which shows that the questionnaire has good measurement validity and reliability.

### Statistical Analysis

SSPS Statistics 21.0 was used for the statistical analysis. After the Shapiro–Wilk normality test, the independent sample *t*-test and one-factor ANOVA were used to analyze the differences in demographic variables. Pearson’s cross-production correlation was used in parents’ education levels, family sports attitude, sports participation, screen time, and children’s BMI. AMOS 21.0 was used to construct the structural equation model and conduct path analysis. The mediation test process proposed by Gerbing and Anderson (1988) was used to explore the mediation effect. First, the independent variable had an impact on the dependent variable, and the regression coefficient was significant. Second, the independent variable affected the intermediary variable, and the regression coefficient was significant. Third, the joint influence of the independent variable and intermediary variable on the dependent variable was significant. The significance level of all indexes was set to α = 0.05.

## Results

### Demographical Variance Analysis of Sports Attitude and Sports Behavior

The results of the independent sample *t*-test (Table 2) show that, in terms of gender, there was no difference in boys’ and girls’ family attitudes toward physical activity (*T* = 0.39, *p* > 0.05). There was no difference in the physical activity behavior between boys and girls (*t* = 0.99, *p* > 0.05). There was no gender difference in screen time (*t* = 1.03, *p* > 0.05). There was no gender difference in infant BMI (*T* = 1.23, *p* > 0.05). The results of analysis of variance showed that: (1) In terms of a mother’s education level, there were differences in the family sports attitude score, children’s sports participation, screen time, and children’s BMI among different mothers’ education levels. Specifically, the scores of the mother with primary school education were lower than those with high school education, while those with high school education were lower than those with a college education or above (*F* = 49.74, *p* < 0.01). There were also significant differences in children’s sports participation between fathers with different education levels (*F* = 18.67, *p* < 0.01). Children with fathers’ education levels had the highest sports participation in college or above, followed by children with fathers’ education levels in middle to high school. There was no significant difference in children’s sports participation between fathers’ education levels in middle and high school and primary school children. The screen time of children was significantly different only between primary school and college or above groups. The screen time of mothers with primary school education was significantly higher than that of college or above groups (*F* = 10.98, *p* < 0.01). In terms of children’s BMI, there were differences between mothers with different education levels. The BMI of children whose mothers had primary school education was significantly higher than that of those from middle school to high school, and the BMI of children whose mothers had middle school to high school education was higher than that of children whose mothers had a college education or above. (2) In terms of fathers’ education levels, there was a significant difference in sports attitude among families with different fathers’ education levels (*F* = 28.39, *p* < 0.01). The score of a father’s education from middle school to high school was significantly higher than that of primary school, and there was no significant difference in the score of family sports attitude when a father’s education was higher than middle school (*p* > 0.05). There were also differences in children’s sports participation among fathers with different education levels. Fathers’ education levels in college and above were significantly higher than those in primary school (*F* = 3.48, *p* < 0.01), while there was no difference between fathers’ education levels in primary school and middle school to high school (*p* > 0.05). In terms of screen time, there was no difference between fathers with college or above education background and those with middle school to a high school education level (*F* = 3.95, *p* < 0.01), while there was no difference between fathers with middle school to high school and primary school education levels (*p* > 0.05). In terms of BMI of children, the father’s education level from middle school to high school was significantly lower than that of primary school, and the father’s education level from university or above was also significantly lower than that of primary school (*F* = 24.35, *p* < 0.01). There was no difference in BMI of children between a father’s education level from middle school to high and those education levels from a college or above (*p* > 0.05). (3) Family structure: there were differences in family sports attitude, sports participation, and screen time among children with different family structures, but there was no significant difference in BMI. Multiple comparisons showed that the scores of family sports attitude in families with parents living together, intergenerational parenting, and three generations living in the same house were significantly higher than those in single-parent families (*F* = 6.02, *p* < 0.01). The results showed that the children’s sports participation of three generations in the same family, intergenerational parenting, and single-parent family was significantly higher than that of a small family (*F* = 152.69, *p* < 0.05). Screen static time per week in families with three generations and parents living together was higher than that in single-parent families (*F* = 1.58, *p* < 0.05).

**TABLE 2:** Demographical variance analysis of sports attitude and sports behavior (*N* = 589).

Variable	Family sports attitude		Sports participation		Screen time		Children’s BMI	
	M	SD	M	SD	M	SD	M	SD
Boy	31.80	7.08	23.25	15.08	5.30	1.89	15.94	1.57
Girls	31.55	8.11	22.06	13.83	5.13	1.96	15.44	1.56
T	0.39		0.99		1.03		1.23	
Primary school (A)	28.04	6.52	18.78	12.66	5.65	1.70	16.87	1.52
Middle school to high school (B)	31.68	8.67	20.14	12.46	5.26	1.91	15.74	1.69
College or above (C)	34.43	6.88	26.19	15.38	4.85	2.03	15.14	1.11
F (mother) Multiple comparison	49.74***		18.67***		10.98***		98.14***	
	C > B; B > A		C > A		A > C		A > B; B > C	
Primary school (a)	28.83	7.87	20.68	13.52	5.42	1.88	16.29	1.82
Middle school to high school (b)	33.34	7.21	23.17	15.24	5.27	1.93	15.60	1.19
College or above (c)	33.58	6.65	24.21	14.62	4.91	1.96	15.57	1.43
F (father) Multiple comparison	28.39***		3.48***		3.95**		24.35***	
	c > a; b > a		c > a		c > a		c > a; b > a	
Single parent (I)	24.45	12.70	35.90	12.84	4.25	2.17	15.23	1.07
Nuclear family (II)	31.94	7.26	13.59	6.93	5.20	1.96	15.92	1.63
Grandparenting (III)	29.56	9.21	34.26	12.77	5.10	2.05	15.47	1.86
Three generations under one roof (IV)	32.44	6.85	32.00	13.38	5.33	1.81	15.88	1.44
F (family structure) Multiple comparison	6.02***		2.29***		1.58**		1.74	
	III > I;		I > IV		IV > I; II > I			

*p < 0.05, **p < 0.01, and ***p < 0.001.

### Correlation Analysis of Family Sports Attitude, Parents’ Education Levels, Sports Participation, Screen Time, and Children’s BMI

Correlation analysis showed (Table 3) that there were significant correlations among the variables. There was a significant negative correlation between family sports attitude, sports participation, parents’ education levels, and children’s BMI, and there was a significant positive correlation between screen time and children’s BMI. Hypothesis H1 of this study was confirmed. There was a significant positive correlation between sports participation and family sports attitude (*r* = 0.32). There was a significant negative correlation between family sports attitude and children’s screen time (*r* = −0.15). There was a significant negative correlation between family sports attitude and children’s BMI (*r* = −0.23).

**TABLE 3:** Analysis of the correlation among family sports attitude, parents’ education levels, sports participation, screen time, and children’s BMI (*N* = 589).

Variables	M	SD	1	2	3	4	5	6
1. Children’s BMI	15.86	1.58	−					
2. Family sports attitude	32.10	8.22	−0.23***	−				
3. Mother’s education level	2.12	0.91	−0.37***	0.19***	−			
4. Father’s education level	1.97	0.86	−0.18***	0.09**	0.55***	−		
5. Sports participation	22.57	14.43	−0.62***	0.32***	0.24***	0.11***	−	
6. One week of screen time	5.12	1.94	0.49***	−0.15***	−0.17***	–0.08	−0.49***	−

*p < 0.05, **p < 0.01, and ***p < 0.001.

### Linear Regression of Family Sports Attitude, Sports Participation, Screen Static Time, and Children’s BMI

The results of linear regression showed that family sports attitude was negatively correlated with children’s BMI. Sports participation was negatively correlated with young children’s BMI. One week of static screen time was positively correlated with children’s BMI. And they all reached a significant level (Table 4).

**TABLE 4:** Linear regression of family sports attitude, sports participation, screen static time, and children’s BMI.

	Family sports attitude			Sports participation			One week of screen time		
	*R* ^2^	β	*P* value	*R* ^2^	β	*P* value	*R* ^2^	β	*P* value
BMI	0.02	−0.14	0.001	0.38	−0.62	<0.001	0.24	0.49	<0.001

### Path Relationship Analysis of Family Sports Attitude, Sports Participation, Screen Static Time, and Children’s BMI

**FIGURE 1:**
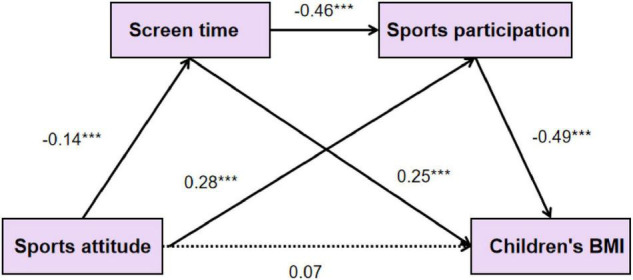
Path relationship between family sports attitude, sports participation, screen static time, and children’s BMI.

The relationship of family sports attitude, sports participation, screen time, and children’s BMI, the mediating effect between screen time, and exercise participation were examined by Wen and Ye mediating effect test procedures (Zhonglin and Baojuan, 2014). Figure 1 shows a structural equation model testing the mediating effect of exercise participation and screen time. The structural equation model contains four potential variables, and the results show that the fitting indexes of the structural equation model are as follows: x2/df = 1.84, df = 1, RMSEA = 0.03, AGFI = 0.98, CFI = 0.99, TLI = 0.99, IFI = 0.99, which indicated that a model can be established well. Figure 1 shows the standardized coefficient in the mediation model diagram, and Table 5 shows the results of indirect effects. As shown in Table 5, the confidence interval of each mediation path does not contain 0, so the mediation effect is significant. Hypothesis H2 in this study is valid. As shown in Figure 1, the direct effect of family sports attitude and children’s BMI in the model was not significant (*r* = −0.04, *p* > 0.05), but the negative prediction effect of family sports attitude and screen time was significant (*r* = −0.14, *p* < 0.001). Family sports attitude and children’s sports participation had a significant positive predictive effect (*r* = 0.25, *p* < 0.001). The BMI of children was predicted negatively by sports participation (*r* = 0.49, *p* < 0.001), while the screen time could predict BMI positively (*r* = 0.25, *p* < 0.001). From the significant levels and the standardized path coefficient b values in the structural model in Figure 1, it is not difficult to find that screen time and sports participation in the model play a mediating role between family sports attitude and children’s BMI, and the mediating role includes three paths: The single mediating effect of screen time and sports participation, and the chain mediating effect of screen time → exercise participation. To verify the mediating effect of sports participation and screen time between family sports attitude and children’s BMI, non-parametric percentile Bootstrap was used to test the significance of mediating effect. The original data were sampled 2,000 times, and a 95% CI was estimated. If the confidence interval did not include 0, the mediating effect was significant. The results in Table 5 show that screen time (95% CI: −0.021, −0.006) and sports participation (95% CI: −0.045, −0.028) played a complete mediating role between family sports attitude and children’s BMI, while screen time → sports participation (95% CI: −0.046, −0.026) played a chain-mediating role. None of the three intervals contain 0, indicating a significant mediating effect. Therefore, hypotheses H1 and H2 of this study have been confirmed.

**TABLE 5:** A statistical table of path weight coefficient (*N* = 589).

No.	Pathway Boot	Standardized mediating effect value (SE)	Bootstrap 95% (CI)
1	Sports attitude→ Screen time→ Children’s BMI	0.01 (0.01)	(−0.021, −0.006)
2	Sports attitude→ Sports participation → Children’s BMI	−0.04 (0.01)	(−0.045, −0.028)
3	Sports attitude→ Screen time→ Sports participation→ Children’s BMI	−0.04 (0.01)	(−0.046, −0.026)

### Difference Test of a Mediation Model in Different Family Structures

Relevant studies found that different family structures in children’s lives can lead to significant differences in children’s sports participation, screen time, and BMI. This study verified whether sports participation and screen time have a stable mediating role in different family structures. Firstly, the mediating effects of children’s sports participation and screen time in nuclear and non-nuclear families were examined respectively. The results showed that the model fitting indexes of nuclear families were: χ2/df = 2.51, AGFI = 0.96, CFI = 0.99, TLI = 0.96, IFI = 0.99, RMSEA = 0.067. The model fitting indexes of non-nuclear families were: χ2/df = 1.67, AGFI = 0.96, CFI = 0.99, TLI = 0.98, IFI = 0.99, and RMSEA = 0.052. The results showed that the fitting indexes of each group were good, which could be compared with different groups of models. Then, the set equivalent model was used to compare the different groups of models, and the results showed that the model had good fitting indexes, and the fitting index difference ΔCFI and ΔTLI values were less than 0.05 (ΔCFI and NTLI were obtained by the difference of TLI and CFI between the nuclear family and the non-nuclear family) (Zhao, 2007). The results show that the mediation model is stable in different family structures.

## Discussion

### Analysis of Influencing Factors of Infant BMI, Family Sports Attitude, Sports Participation, and Screen Time

**FIGURE 2:**
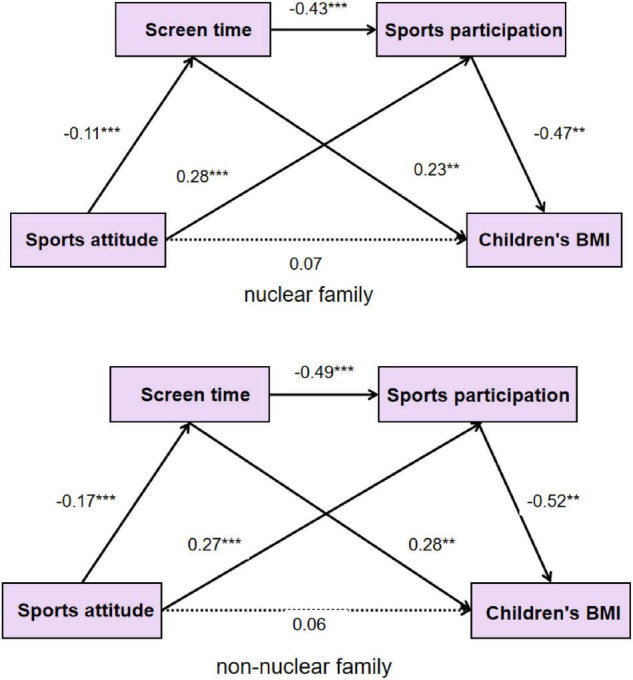
Path relationship between family sports attitude, sports participation, screen static time, and children’s BMI in different family structures.

This study found no gender differences in children’s BMI, family sports attitude, weekly sports participation, and static screen time, which is consistent with the results of the Xinran Shi’s study (Shi et al., 2021). At the same time, this study found that children with different family structures have significant differences in sports attitudes, screen time, and sports participation (Figure 2). Further analysis found that children with single-parent family structure scored the lowest in family sports attitude, and there were significant differences in living with their parents, grandparenting, and three generations in the same family. Previous studies showed that children living in the single-parent family structure were more likely to have physical and mental health problems than those who live in a two-parent family structure (Ledoux et al., 2002; Wang et al., 2012). Quarmby et al. (2011) confirmed that the lower socioeconomic status of single-parent families compared with two-parent families partly explains the poorer physical health of their children. Compared with European and American countries, the major feature of Chinese family structure is grandparenting. There are different conclusions about the advantages and disadvantages of grandparenting. Opponents believe that grandparents are prone to develop bad health habits due to poor health habits and lack of nutrition knowledge. Supporters believe that grandparents’ love for their grandchildren, abundant time and energy investment in a company, education, and nurturing experience are all beneficial to the healthy development of grandchildren (Lu et al., 2020). In this study, we found that the sports participation of three-generation families was higher than that of families living with parents only, which may be related to the time advantage of grandparents to pay attention to and accompany their children’s activities. Other researchers have suggested that the three-generation family structure is more conducive to the formation of healthy concepts in young children, as the role of family bonding is played by grandparents living in the same family.

In addition, this study found that the higher the mother’s education level, the lower the children’s BMI. Qian et al. conducted a study on Chinese students aged 8–10 and found that the group whose parents have a low educational level has a low score in dietary habits and nutritional knowledge, while dietary habits are significantly correlated with BMI (Han et al., 2016). Han Hui et al. found a significant correlation between children’s food preference risk and their mothers’ education levels, and the higher the mother’s education level, the lower the risk of children’s food preference risk (Qian et al., 2018). The study also found that parents’ education levels were negatively correlated with children’s screen static time. Researchers also found that the daily screen time of children whose parents have a college education level or above was significantly lower than that of children whose parents have a primary school education level or below. The study also found that children whose parents had college education levels spend more time on moderate-intensity activity than children whose parents had middle school education levels or below (Wärnberg et al., 2021). The results of this study are also consistent with those of other studies (Pons et al., 2020). So, the results of this study are not only limited to Chinese children but also consistent with those of Spanish and Caucasian children; the results showed that the educational level of the parents was related to the health habits of children (Duch et al., 2013; Carson et al., 2017; Monserrat et al., 2020; Yañez et al., 2020). Wang et al. (2015) found in their research that the concept of exercise will gradually internalize into self-identity with the improvement of the education level, and the influence of the education level on physical exercise behavior will be increasingly enhanced. Therefore, parents’ education levels have a profound influence on family physical exercise.

### Family Sports Attitude and Children’s BMI

There is a significant negative correlation between family sports attitude and children’s BMI, which is consistent with the previous research results of Gao Yan et al. A large number of studies have shown that the decisions of children’s sports participation at the beginning, middle, and end are related to the environment. Human behavior is formed by the interaction between individuals and the environment. The environmental models that affect human development include micro, medium, and macro-environmental models. Family is an important part of the microenvironment, which plays a key role in the process of children’s growth (Sun et al., 2006; Jian et al., 2014; Yang et al., 2014). Family is not only the “gatekeeper” of children’s sports activities but also the key factor of children’s screen time duration (Strasburgerand Council on Communications and Media American Academy of Pediatrics, 2010). Effective intervention measures aimed at family factors can affect children’s physical condition. Therefore, a better understanding of the influence of family sports attitude on children’s physical health is helpful to reduce the incidence of childhood obesity and malnutrition caused by family factors. Previous studies have shown that the influence of family, parents, and elders on children’s sports behavior can be divided into two aspects: the support of elders for children’s sports and the shaping of the role model of elders (Clark et al., 2011; Morgan et al., 2012; Rhodes and Quinlan, 2014). All the above results indicate the importance of family sports attitude in children’s physical health among the influencing factors of the family on children’s physical health, and family should be regarded as the implementation focus of children’s physical health intervention.

### Chain-Mediated Effects of Children’s Screen Time and Exercise Participation

In this study, screen time and sports participation were used as mediating variables to construct a mediating model of family sports attitudes affecting children’s BMI. The mediating effect of sports participation and screen time is significant, which indicates that they played an important role in family sports attitudes in interpreting children’s BMI. Fan Huiying et al. found that family support has a significant impact on children’s physical activities, and family as the starting point of children’s sports participation there was the strongest correlation between their support for children’s physical activity attitude and children’s sports (Craggs et al., 2011; Yao and Rhodes, 2015; Pyper et al., 2017; Hutchens and Lee, 2018). So, family sports attitudes play an important role in young children’s sports participation and static screen time limitation. Parents, as the first teachers of children’s physical education class, will have a positive impact on children’s sports by setting an example in sports participation and supporting the concept of physical education, whereas the negative performance of family physical education attitude and behavior will have a negative impact on children. Reilly et al. (2014) found that Scottish children’s physical fitness declined due to insufficient sports participation in recent years. One important and direct reason is that parent**s** have a negative attitude toward sports participation (Reilly et al., 2014). Researchers found that the longer children’s moderate-intensity activity time was, the shorter their screen time was, which was related to children’s family sports cognition (Wärnberg et al., 2021). This view was consistent with the results of this study. Parents’ support for children’s sports can improve their children’s sports participation, while children’s sports exercise is negatively correlated with children’s BMI (Brown et al., 2016). Sports attitude can influence children’s BMI through sports participation. Another important factor affecting BMI is sedentary behavior, which includes screen time, homework time, and so on. Related studies have found that sedentary behavior mainly based on screen time has a negative impact on health problems such as overweight and myopia in adolescents and children. Moreover, the study found that family and peer support had a significant impact on sedentary screen time, and parents’ support for children’s physical activity was negatively correlated with screen time (Biddle, 2007; Vander Ploeg and Hillsdon, 2017). It can be seen that the family environment constitutes an external driving force for children’s screen time, which is mainly manifested in exercise support and participation. When family members form a state of “high screen time—low exercise time,” children’s screen time can be invisibly increased, thus leading to the increase of their BMI. This indicates that the mediating effect of screen time and exercise participation in this research model is feasible, and it can be seen from Figure 1 that the mediating effect is completely mediating. To some extent, this result explained the influence mechanism of family sports attitude on children’s BMI and provided evidence for the intervention of family factors in children’s physical health development.

### Differences in Mediating Effects of Screen Time and Exercise Participation Among Different Family Structures

Screen time and exercise participation were fully mediating effects in different family structures, and the chain-mediated effects held in both nuclear and non-nuclear families. In the family structure, the nuclear family is the family composed of husband and wife and unmarried children, while the non-nuclear family includes three generations living under the same family, single parent, and grandparenting families. This study found that the total mediating effect of screen time and exercise participation in non-nuclear families was higher than those in nuclear families, which may be related to the higher proportion of three generations living in the same family in non-nuclear families. The family structure of three generations under one roof is the most abundant and complete. Children grow up in this environment with both parents and grandparents. The study found that grandparents had more time and energy to spend with and participate in children’s activities than busy parents. However, the lack of parents’ company and upbringing may result in children’s bad health behavior habits due to the lack of scientific sports knowledge, nutrition knowledge, and poor health concept of their grandparents (Huang and Chen, 2007). Therefore, both parents and grandparents play important roles in the development of healthy behaviors in young children. In this study, there were significant differences in children’s family sports attitude, sports participation, and screen time among different family structures, but there was no significant difference in the mediating role model of sports participation and screen time. The results indicated that the three factors of family sports attitude, sports participation, and screen time were different due to the influence of family structure variables, but the chain-mediating effect of sports participation and screen time in the influence mechanism of family sports attitude on children’s BMI had internal similarities among different children’s family structures.

## Conclusion and Prospect

### Conclusion

There are significant differences in children’s family sports attitude, sports participation, screen time, and BMI with different parents’ education levels. Specifically, the higher the parents’ education levels, the higher the family sports attitude score, the better the sports participation, and the lower the screen time. Family sports attitude is positively correlated with sports participation and a mother’s education level and negatively correlated with children’s screen static time and BMI. Sports participation and screen time play a completely mediating role in the influence path of family sports attitude on children’s BMI. The mediating mechanisms of sports participation and screen time were not affected by family structure (nuclear and non-nuclear families), but the total mediating effect of sports participation and screen time was higher in non-nuclear families than in nuclear families. The results of this study will provide a useful reference for teachers and parents to control children’s physical health and the influencing factors of children’s physical health research in the future.

### Limits and Prospects

This study identified the mediating role of sports participation and screen time. The structural equation model of the relationship between the attitude of plus family and children’s BMI was constructed and verified. And the study still has the following limitations and suggestions for future research:

(1) Among the family influencing factors of children’s BMI, this study focused on examining the mediating effects of children’s sports participation and static screen time.
(2) This study was a cross-sectional study. Future research can further support the conclusion of this study by using the exercise tracking method and providing direct evidence for variable causality.

## Data Availability Statement

The original contributions presented in the study are included in the article/supplementary material, further inquiries can be directed to the corresponding author/s.

## Ethics Statement

The studies involving human participants were reviewed and approved by the Ethics Committee of Sports School of Southwest University in China. Written informed consent to participate in this study was provided by the participants’ legal guardian/next of kin.

## Author Contributions

CP and LJ carried out the protocol and the questionnaire survey. YL recruited the survey respondents. ZT, LH, and YJ undertook the statistical analysis and graphical representation of the data. LW revised the draft. All authors participate in the completion of the study and contributed to and approved the final manuscript.

## Conflict of Interest

The authors declare that the research was conducted in the absence of any commercial or financial relationships that could be construed as a potential conflict of interest.

## Publisher’s Note

All claims expressed in this article are solely those of the authors and do not necessarily represent those of their affiliated organizations, or those of the publisher, the editors and the reviewers. Any product that may be evaluated in this article, or claim that may be made by its manufacturer, is not guaranteed or endorsed by the publisher.

## Footnotes

1 Research Group of Chinese National Physical Fitness Monitoring System, Department of Science and Education, General Administration of Sport of China Study on Chinese National Physical Fitness Monitoring System [M]. Beijing: Beijing Sport.
